# Remodeling of Cancer-Specific Metabolism under Hypoxia with Lactate Calcium Salt in Human Colorectal Cancer Cells

**DOI:** 10.3390/cancers13071518

**Published:** 2021-03-25

**Authors:** Keun-Yeong Jeong, Jae-Jun Sim, Min Hee Park, Hwan Mook Kim

**Affiliations:** 1 Metimedi Pharmaceuticals Co., Research Center, 263 Central-Ro, Yeonsu-Gu, Incheon 22006, Korea; genesis0804@hanmail.net (J.-J.S.); pmh1880@hanmail.net (M.H.P.); 2 Gachon Institute of Pharmaceutical Sciences, Gachon University 191 Hambangmoe-Ro, Yeonsu-Gu, Incheon 21936, Korea

**Keywords:** colorectal cancer, lactate calcium salt, anaerobic glycolysis, hypoxia, lactate dehydrogenase B, hypoxia-inducible factor

## Abstract

**Simple Summary:**

This study was to prove the changes in cancer-specific metabolism caused by the introduction of lactate calcium salt into human colorectal cancer cells from the viewpoint of remodeling in anaerobic glycolysis and the tricarboxylic acid cycle under hypoxia. An influx of lactate calcium salt-induced enzymatic activation of lactate dehydrogenase B reacting to lactate followed by the decrease in the transcriptional activation of hypoxia-inducible factor-1α to suppress the expression of the oncogenes. Thereby, it was possible to induce anti-cancer effects on the colorectal cancer xenograft animal model.

**Abstract:**

Hypoxic cancer cells meet their growing energy requirements by upregulating glycolysis, resulting in increased glucose consumption and lactate production. Herein, we used a unique approach to change in anaerobic glycolysis of cancer cells by lactate calcium salt (CaLac). Human colorectal cancer (CRC) cells were used for the study. Intracellular calcium and lactate influx was confirmed following 2.5 mM CaLac treatment. The enzymatic activation of lactate dehydrogenase B (LDHB) and pyruvate dehydrogenase (PDH) through substrate reaction of CaLac was investigated. Changes in the intermediates of the tricarboxylic acid (TCA) cycle were confirmed. The cell viability assay, tube formation, and wound-healing assay were performed as well as the confirmation of the expression of hypoxia-inducible factor (HIF)-1α and vascular endothelial growth factor (VEGF). In vivo antitumor effects were evaluated using heterotopic and metastatic xenograft animal models with 20 mg/kg CaLac administration. Intracellular calcium and lactate levels were increased following CaLac treatment in CRC cells under hypoxia. Then, enzymatic activation of LDHB and PDH were increased. Upon PDH knockdown, α-ketoglutarate levels were similar between CaLac-treated and untreated cells, indicating that TCA cycle restoration was dependent on CaLac-mediated LDHB and PDH reactivation. CaLac-mediated remodeling of cancer-specific anaerobic glycolysis induced destabilization of HIF-1α and a decrease in VEGF expression, leading to the inhibition of the migration of CRC cells. The significant inhibition of CRC growth and liver metastasis by CaLac administration was confirmed. Our study highlights the potential utility of CaLac supplementation in CRC patients who display reduced therapeutic responses to conventional modes owing to the hypoxic tumor microenvironment.

## 1. Introduction

Cancer cells have characteristic, atypical metabolic requirements that lead to changes in cell physiology favorable for uncontrolled cell growth [1]. Specifically, cancer progression is triggered by upregulated glycolysis, which is facilitated by large amounts of glucose entering the tumor cells through glucose transport channels (GLUT) [2]. In normal cells, glucose is converted into pyruvate via multiple glycolytic reactions, and this pyruvate enters the mitochondria, where it is converted into acetyl coenzyme A via pyruvate dehydrogenase (PDH) [3]. Subsequently, adenosine triphosphate is synthesized via oxidative phosphorylation in the mitochondria [3,4]. However, in hypoxic cancer cells, active pyruvate dehydrogenase kinase (PDK) inhibits PDH, resulting in the uncoupling of the tricarboxylic acid (TCA) cycle and stimulation of lactate dehydrogenase A (LDHA). This leads to the constitutive production of lactate in cancer cells [4,5].

Anaerobic glycolysis is a common metabolic pathway adopted by hypoxic cancer cells and is regulated by hypoxia-inducible factor (HIF)-1α [2]. HIF-1α transcription stimulates the expression of LDHA and monocarboxylate transporter, which is required for metabolic reprogramming and the production and export of lactate respectively [6]. Moreover, lactate serves as the primary metabolite associated with chronic glucose utilization by cancer cells and facilitates its self-sufficient mode of metabolism and development of an acidic tumor microenvironment to aid invasion and metastasis [7]. To date, various studies have sought to inhibit or suppress the production of lactate in cancer cells [2,8]. Specifically, mechanisms have been proposed that reduce lactate production and accumulation by inhibiting LDHA with oxamate, or by increasing PDH activity with dichloroacetate, which directs glycolytic carbon flux and interrupts lactate-related metabolism [9,10]. However, considering that lactate is also required for normal cell metabolism, the toxic effects induced by these strategies must also be considered [11,12]. Therefore, developing a novel strategy to facilitate the switch to a benign metabolism that reduces lactate production may prove to be an important tool for modulating cancer metabolism.

Oxidative consumer cells maintain an equilibrium between intracellular pyruvate and lactate by activating LDHA or LDHB [2,6]. LDHB, an isoform of LDHA, favors the conversion of lactate into pyruvate, thereby supporting oxidation during the TCA cycle [13]. In cancer cells, the LDHB enzyme is relatively downregulated in hypoxic conditions [14]; however, its role in hypoxic cancer metabolism has not yet been elucidated. The cause is probably the fact that there is no suitable method to enhance LDHB activation under hypoxia currently. Thus, finding novel strategies aimed at LDHB in hypoxic cancer cells is warranted.

Lactate calcium salt (CaLac) is a small molecule composed of one calcium and two lactate ions and is characterized as a weak electrolyte with a neutral charge [15]. The characteristic of CaLac would have a chance for it to be able to readily cross the cell membranes in a bound form even without specific transporter. CaLac can serve as a substrate to stimulate LDHB and influences intracellular calcium accumulation. In the current study, we focused on the unique metabolic changes in anaerobic glycolysis following the CaLac influx into human colorectal cancer (CRC) cells exposed to hypoxia.

## 2. Materials and Methods

### 2.1. Cell Lines and Culture Conditions

All cell lines were purchased from the American Type Culture Collection (ATCC; Manassas, VA, USA). The human CRC cell lines, HCT116 and HT29, were grown in Roswell Park Memorial Institute (RPMI) 1640, or glutamine-free RPMI1640 medium (Welgene, Daegu, Korea), supplemented with 10% fetal bovine serum (Welgene) and 1% penicillin/streptomycin (Welgene). Human umbilical vein endothelial cells (HUVECs) (ATCC, Manassas, VA, USA) were maintained using the EGM-2 bullet kit (Lonza, Basel, Switzerland). Cells were grown at 37 °C under hypoxic conditions, with 1% oxygen, 5% CO_2_, and 94% nitrogen (COY Laboratory, Grass Lake Charter Township, Grass Lake, MI, USA).

### 2.2. Reagents

CaLac was purchased from Sigma-Aldrich (St. Louis, MO, USA) and dissolved in distilled water. The solution was stored at 4 °C until use.

### 2.3. Measurement of Intracellular Calcium and Lactate

Calcium concentrations were measured using a confocal laser scanning microscope (Leica, Heidelberg, Germany). Cultured HCT116 and HT29 cells were loaded with 10 μM Fluo-3/AM and 1 μL 25% Pluronic F-127 (Sigma-Aldrich) dissolved in dimethyl sulfoxide (Sigma-Aldrich) and incubated for 30 min at 37 °C. After loading fluorescence probes, CaLac was added, and images were acquired. Fluo-3/AM was excited at 488 nm and emitted fluorescence was measured at 515 nm. The lactate concentration was determined using the lactate assay kit (Abcam, Cambridge, England). CRC cells (5 × 10^5^) were seeded in 6-well plates and grown overnight. Subsequently, the cells were treated with 2.5 mM CaLac for 24 h. Whole-cell extracts were prepared using sonication, and intracellular lactate was measured using quantitative analysis, according to the kit protocol (Abcam).

### 2.4. Quantification of Enzyme Levels

The cell culture medium was supplemented with CaLac, and cultures were incubated for 24 h. 2 × 10^6^ cells were then sonicated. LDHB, citrate synthase, isocitrate dehydrogenase, and prolyl hydroxylase (PHD) levels were quantified using each assay kit (Biovision, Milpitas, CA, USA), according to the manufacturer’s instructions after treatment with CaLac for 24 h.

### 2.5. Immunocytochemistry

CRC cells were cultured on bio-coated coverslips (BD bioscience, San Jose, CA, USA) and fixed using 4% paraformaldehyde. They were then incubated with primary antibodies against the following proteins for 15 h: lactate dehydrogenase B (LDHB, 1:200, Abcam) and phospho-pyruvate dehydrogenase (pPDH, 1:200, Abcam). Subsequently, the cells were incubated with an anti-rabbit secondary biotinylated antibody (1:5000, Abcam), and the proteins were visualized with streptavidin conjugated to fluorescein (Vector Laboratories, Burlingame, CA, USA). Fluorescence was measured using a confocal laser scanning microscope (Leica). Signal intensities were quantified and analyzed using the Xenogen Imaging System (IVIS^®^ 100 series, Caliper Life Science, Waltham, MA, USA).

### 2.6. Western Blotting

Cytosolic protein lysates and nuclear fractions were prepared using RIPA buffer containing a protease inhibitor cocktail (Roche, Basel, Switzerland) or nuclear extraction kit (Abcam) respectively. Their concentrations were measured using the BCA assay kit (Thermo Scientific, Waltham, MA, USA). Proteins were separated on 6–12% sodium dodecyl sulfate-polyacrylamide gels by electrophoresis and transferred onto polyvinylidene fluoride membranes. The blots were incubated with the following primary antibodies: anti-LDHB (1:1000, Cell Signaling, Danvers, MA, USA), anti-PDH (1:1000, Cell Signaling), anti-actin (1:10,000, Cell Signaling), anti-HIF-1α (1:1000, Cell Signaling), anti-VEGF-A (1:1000, Cell Signaling), and anti-lamin B (1:1000, Cell Signaling). Subsequently, secondary antibodies were used to detect specific proteins (1:10,000, Cell Signaling).

### 2.7. Metabolite Assay

The cell culture medium was supplemented with CaLac, and cultures were incubated for 24 h; 2 × 10^6^ cells were then sonicated and passed through a 10 kDa centrifuge filter (Biovision) at 12,000× *g* for 10 min. Citrate, pyruvate, and α-ketoglutarate in the filtrate were quantified using each assay kit (citrate and α-ketoglutarate assay kit: Biovision; pyruvate assay kit: Abcam), following the manufacturer’s protocol.

### 2.8. Small Interfering RNA (siRNA) Transfection

Specific siRNAs were synthesized by the Bioneer Corporation (Daejeon, Korea). The sequences of the siPDH primer pairs are as follows: sense: 5′-CUGUACGCCGAAUGGAGUUdTdT-3′; anti-sense: 5′-AACUCCAUUCGGCGUACAGdTdT-3′. The siRNAs were transfected into CRC cells using RNAiMAX (Invitrogen, Carlsbad, CA, USA).

### 2.9. RNA Extraction and Quantitative Real-Time Polymerase Chain Reaction (PCR)

Total RNA was extracted from whole cells using TRIzol (Invitrogen, Carlsbad, CA, USA) and subsequently used to synthesize cDNA. Quantitative real-time polymerase chain reaction (PCR) was performed using the SYBR Premix ExTaq system (Takara, Shiga, Japan) and Stratagene Mx3005P detector (Agilent Technologies, Santa Clara, CA, USA). Table 1 shows the sequences of the primer pairs used.

### 2.10. Cell Viability Assay

Colorectal cancer cells were cultured in a 96-well plate (3 × 10^3^ cells/well) for 24 h, and then treated with different concentrations of CaLac (0.5, 1, and 2.5 mM) for 24 h at 37 °C. Then, 10 μL of 3-(4,5-dimethylthiazol-2-yl)-2,5-diphenyltetrazolium bromide was added to each well, and the cells were incubated at 37 °C for 1 h in a humidified environment containing 5% CO_2_. After the media was discarded, 200 μL of dimethyl sulfoxide (Cell Signaling Technology, Danvers, MA, USA) was added to each well. The absorbance was read at 570 nm using a microplate reader (iMark Microplate Absorbance Reader, Bio-Rad, Hercules, CA, USA).

### 2.11. Tube Formation Assay

Colorectal cancer cells were cultured overnight and subjected to CaLac treatment for 24 h. The conditioned medium was collected using a filter unit (Millipore, Burlington, MA, USA) and centrifuged at 12,000× *g* for 10 min. HUVECs were seeded on reduced growth factor-basement membrane extract with the conditioned medium in 96-well plates. Tube formation was visualized using fluorescence microscopy (Olympus, Tokyo, Japan).

### 2.12. Wound-Healing Assay

For the wound-healing assay, a culture insert consisting of two reservoirs separated by a 500 μm thick wall was used. HCT116 cells were seeded into a two-chamber (100 μL of 4 × 10^5^ cells/mL) culture insert. After 24 h incubation, the wall was gently removed creating a gap of ~500 μm. Cells were then allowed to migrate into the bared areas for 12 h. The cell images were captured using the JuLi Br, Live cell analyzer (NanoEnTek Inc., Seoul, Korea).

### 2.13. Xenograft Animal Model

All experiments were performed by conforming with the guidelines established by the Institutional Animal Care and Use Committee at Gachon University (IACUC-LCDI-2019-0102, approval date: 11 March 2019). Twenty-eight male Balb/c nude mice (5 weeks of age) were purchased from the Charles River Breeding Laboratories (Wilmington, MA, USA). All animals were maintained on a 12 h light/dark cycle (light on, 08:00) at 22–25 °C, with free access to food and water. To investigate the antitumor activity of CaLac in vivo, a therapeutic strategy was devised to investigate its effect on the heterotopic and metastatic xenograft CRC animal model. Twenty mice were used for establishing a heterotopic xenograft model using HCT116 cells (5 × 10^6^) resuspended in 100 μL of phosphate-buffered saline. The HCT116 cells were injected subcutaneously into the left flank of mice. Once the tumors reached a diameter of 8 mm, 10 mice were randomly divided into the untreated group, and 10 mice were divided into the CaLac-treated group. Five mice were reared per cage until the end of the experiment; 20 mg/kg of CaLac was subcutaneously injected daily for 21 days. Tumor growth was monitored three times per week. Six mice were used for establishing the metastatic xenograft model using 5 × 10^6^ of HCT116 cells resuspended in 100 μL phosphate-buffered saline (one injection volume). Mice were anesthetized using 2% isoflurane, and then a small incision in the left abdominal flank was made, and the spleen was exteriorized for the intra-splenic injection. The cell suspension was injected into the spleen using a 30-gauge needle. To prevent tumor cell leakage and bleeding, a cotton swab was held over the site of injection for 1 min. The injected spleen was placed within the abdomen, and the incision was sutured using 6-0 black silk. After the convalescence stage (7 days), three mice were randomly divided into the untreated group, and three mice were divided into the CaLac-treated group. Mice were reared four per cage until the end of the experiment; 20 mg/kg of CaLac was subcutaneously injected daily for 21 days. To harvest tumors for histological and molecular biology analysis, carbon dioxide inhalation was used for euthanasia at the end of the experiments. A participant flowchart was indicated in the Figure S1.

### 2.14. Mouse Fluorine-18-Fluorodeoxyglucose Positron Emission Tomography/Computed Tomography (^18^F-FDG-PET/CT) Scanning

At the end of CaLac administration in heterotopic xenograft mice, fluorine-18-fluorodeoxyglucose positron emission tomography/computed tomography (^18^F-FDG-PET/CT) analysis was performed using 5 mice per group randomly (untreated and CaLac-treated groups, total 10 mice). Mice were anesthetized using 2% isoflurane, after which 100 μL of sterilized saline was injected subcutaneously to ensure adequate hydration. ^18^F-FDG was administered via the tail vein. One hour later, mice were anesthetized and placed in the PET scanner’s gantry. PET data were acquired for 15 min, using an Inveon small-animal PET/CT imaging system (Preclinical Solutions, Siemens Healthcare Molecular Imaging, Knoxville, TN, USA).

### 2.15. Immunohistochemistry

Tumor tissues were fixed with 10% neutral-buffered formalin for 24 h and embedded in paraffin. Paraffin-embedded 5-μm sections were deparaffinized and rehydrated, washed in distilled water, and subjected to heat-mediated antigen retrieval. Endogenous peroxidase activity was quenched by treatment with 3% H_2_O_2_ for 30 min, and sections were washed with phosphate-buffered saline for 5 min. The sections were blocked for 1 h with a blocking agent (Invitrogen) and incubated overnight with antibodies against LDHB or PDH (1:1000, Cell Signaling) at 4 °C. The sections were then incubated at room temperature with a biotinylated secondary antibody (1:500, Vector Laboratories, Burlingame, CA, USA) for 1 h. The slides were washed and treated with 3,3′-diaminobenzidine tetrahydrochloride (Vector Laboratories). Sections were then counterstained with hematoxylin and mounted in aqua mount (Vector Laboratories).

### 2.16. Immunofluorescence

Cryo-sectioned tumor tissues (5 µm) were incubated overnight with a primary antibody against CD31 (1:200, Abcam). The sections were then incubated with an anti-rabbit secondary biotinylated antibody (1:1000, Vector Laboratories) and visualized with streptavidin conjugated to fluorescein (Vector Laboratories). Fluorescence was measured with a confocal laser scanning microscope (Leica).

### 2.17. Statistical Analysis

All data have been presented as mean ± standard deviation. Significance was analyzed using Student’s *t*-test or one-way analysis of variance (ANOVA), based on the normality of the data. *p* < 0.05 was considered significant. All statistical analyses were performed using Sigma Stat (ver. 3.5, Systat Software Inc., San Jose, CA, USA).

## 3. Results

### 3.1. The Direct Effect of Lactate Calcium Salt (CaLac) Influx into Colorectal Cancer (CRC) Cells on Anaerobic Glycolysis

**Figure 1:**
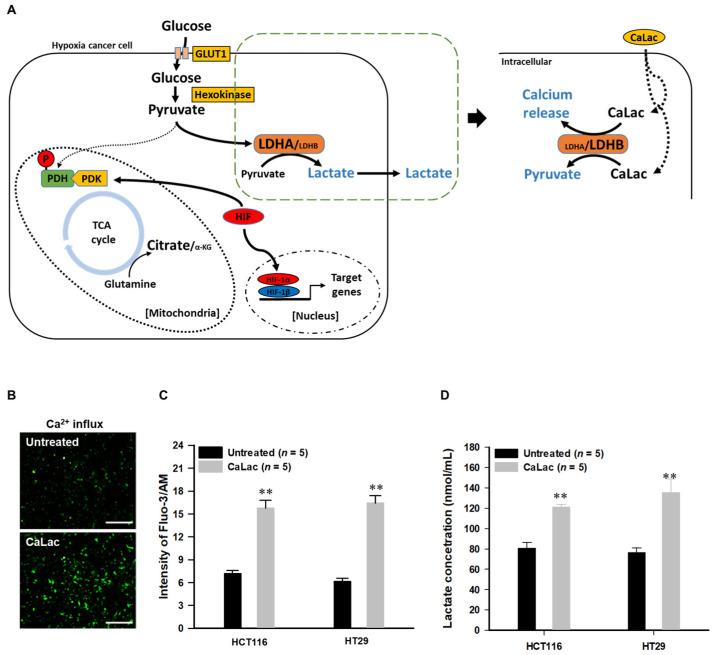
Confirmation of lactate calcium salt (CaLac) influx into colorectal cancer (CRC) cells under hypoxia. (**A**) Schematic illustration of the direct effects of CaLac on anaerobic glycolysis under hypoxia. A large amount of glucose is influx into hypoxic cancer cells and converted to pyruvate via hexokinase [16]. The pyruvate is converted to lactate through lactate dehydrogenase A (LDHA) and outfluxed from the cancer cell. However, activation of LDHB by CaLac reduces the action of LDHA (Figure S2) and leads to an increase in pyruvate from CaLac. (**B**) Immunofluorescence calcium imaging following 2.5 mM CaLac treatment in HCT116 cells. Scale bars, 200 μm (**C**) Quantitative intracellular calcium levels following 2.5 mM CaLac treatment in CRC cells; 2 × 10^6^ cells were analyzed respectively. (**D**) Intracellular lactate levels following 2.5 mM CaLac treatment in CRC cells. 2 × 10^6^ cells were analyzed respectively. ** *p* < 0.001 vs. Untreated. Results are represented as mean ± standard deviation.

**Figure 2:**
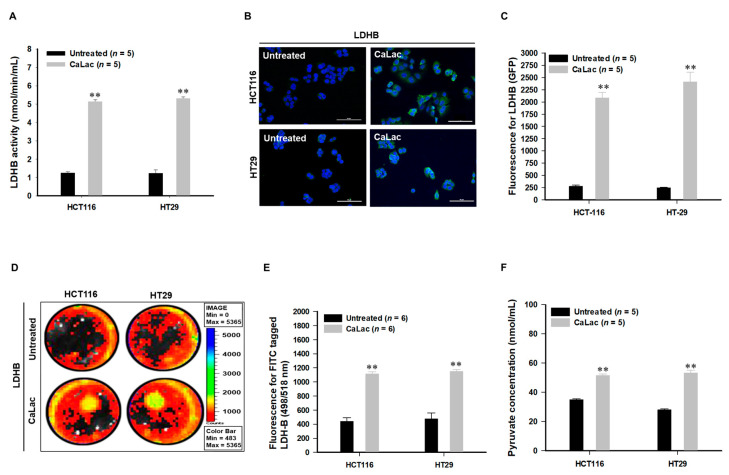
Changes in anaerobic glycolysis caused by lactate calcium salt (CaLac) influx into colorectal cancer (CRC) cells CaLac acts as a substrate for the reactivation of lactate dehydrogenase B (LDHB) and induced the conversion of lactate into pyruvate. (**A**) Confirmation of LDHB activity following 2.5 mM CaLac treatment in CRC cells. 2 × 10^6^ cells were analyzed respectively. (**B**) Confocal micrographs of LDHB reactivation in CRC cells following 2.5 mM CaLac treatment. Scale bars, 100 μm. (**C**) Quantitative analysis for GFP activity using Image J. (**D**,**E**) Representative pictures by IVIS for FITC-tagged LDHB expression following 2.5 mM CaLac treatment in CRC cells. And, quantitative analysis of LDHB expression. (**F**) Intracellular pyruvate levels following 2.5 mM CaLac treatment; 2 × 10^6^ cells were analyzed respectively. ** *p* < 0.001 vs. Untreated. Results are represented as mean ± standard deviation.

Figure 1A is a schematic representation of the activation of LDHB due to CaLac influx into CRC cells under hypoxia. CaLac influx caused an increase in intracellular calcium (Figure 1B,C) and lactate (Figure 1D). Then, LDHB was significantly activated following CaLac treatment (Figure 2A–E). In the analysis of LDHB activity through cytoplasmic extraction, LDHB showed significant enzymatic activity by CaLac (Figure 2A). It was confirmed that the fluorescence intensity of LDHB increased in the cell imaging (Figure 2B,D). A separate fluorescence picture for Figure 1B is shown in Figure S3. Quantitative values corresponding to fluorescence images indicate a significant increase in LDHB expression in CRC cells (Figure 2C,E). It is suggested that the lactate ions of CaLac act as a substrate for LDHB in CRC cells under hypoxia. This was further supported by increased intracellular pyruvate levels following CaLac treatment (Figure 2F). Considering pyruvate could also be increased following a large influx of glucose into cancer cells under hypoxia, the pyruvate level was also compared following the knockdown of LDHB (Figure S4). The results show pyruvate levels were similar between CaLac-treated and untreated cells, indicating that pyruvate production was dependent on CaLac-mediated LDHB reactivation. Moreover, there was no effect of the CRC cell viability by LDHB knockdown. (Figure S4).

### 3.2. Restoration of the Tricarboxylic Acid (TCA) Cycle under Hypoxia Subsequent to Increased Intracellular Calcium and Pyruvate Levels

**Figure 3:**
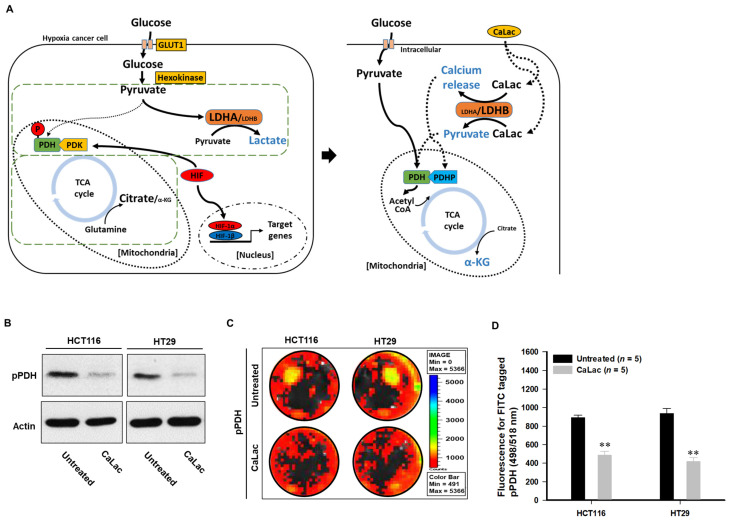
Induction of tricarboxylic acid (TCA) cycle through pyruvate and dissociated calcium under hypoxia. Dephosphorylation and activation of pyruvate dehydrogenase (PDH) were stimulated by calcium., and pyruvate was utilized for TCA cycle restoration, leading to an increase in a-ketoglutarate (α-KG). (**A**) Schematic illustration of TCA cycle restoration by pyruvate and dissociated calcium. PDH activity is inhibited by kinase activity of pyruvate dehydrogenase kinase (PDK) under hypoxia [18], and the inactivation of PDH can be confirmed by an increase in phospho-PDH (pPDH) [19]. However, the increase of intracellular calcium upregulates phosphatase activity followed by PDH activation. Then, the TCA cycle is coupled, and the converted pyruvate is utilized in the metabolic process of the active TCA cycle. (**B**) Decreased phospho-PDH (pPDH) at the protein level following 2.5 mM CaLac treatment in CRC cells. Uncropped blots were indicated in Figure S5 (**C**,**D**) Quantitative analysis of pPDH expression in CRC cells using FITC-tagged pPDH. ** *p* < 0.001 vs. Untreated. Results are presented as mean ± standard deviation.

**Figure 4:**
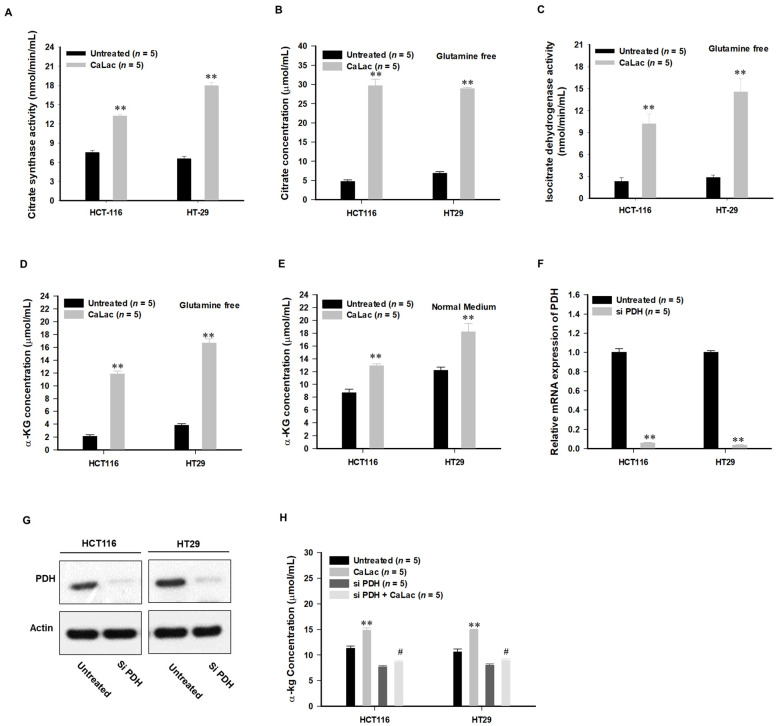
Induction of tricarboxylic acid (TCA) cycle through pyruvate and dissociated calcium under hypoxia. Dephosphorylation and activation of pyruvate dehydrogenase (PDH) were stimulated by calcium, and Pyruvate was utilized for TCA cycle restoration, leading to an increase in a-ketoglutarate (α-KG). (**A**) Schematic illustration of TCA cycle restoration by pyruvate and dissociated calcium. PDH activity is inhibited by kinase activity of pyruvate dehydrogenase kinase (PDK) under hypoxia, and the inactivation of PDH can be confirmed by an increase in phospho-PDH (pPDH). However, the increase of intracellular calcium upregulates phosphatase activity followed by PDH activation. Then, the TCA cycle is coupled, and the converted pyruvate is utilized in the metabolic process of the active TCA cycle. (**B**) Decreased phospho-PDH (pPDH) at the protein level following 2.5 mM CaLac treatment. (**C**,**D**) Quantitative analysis of pPDH expression in CRC cells using FITC-tagged pPDH. (**A**) Comparison of citrate synthase activity; 2 × 10^6^ cells were analyzed, respectively. (**B**) Citrate concentration under glutamine-free culture conditions following 2.5 mM CaLac treatment in CRC cells; 2 × 10^6^ cells were analyzed, respectively. (**C**) Isocitrate dehydrogenase activity under glutamine-free culture conditions following 2.5 mM CaLac treatment in CRC cells; 2 × 10^6^ cells were analyzed, respectively. (**D**,**E**) α-KG concentration under glutamine-free or normal medium culture conditions following 2.5 mM CaLac treatment in CRC cells; 2 × 10^6^ cells were analyzed, respectively. (**F**,**G**) Pyruvate dehydrogenase (PDH) knockdown in CRC cells following siPDH transfection. Uncropped blots for Figure 4G were indicated in Figure S9. (**H**) Confirmation of relationship between PDH knockdown and α-KG production following 2.5 mM CaLac treatment in CRC cells; 2 × 10^6^ cells were analyzed, respectively. ** *p* < 0.001 vs. Untreated. # *p* < 0.001 vs. CaLac. Results are presented as mean ± standard deviation.

Lactate in CaLac acts as a substrate for LDHB, and then calcium, the other component of CaLac, was simultaneously dissociated (Figure 1B,C). Therefore, the role in an increase in intracellular calcium was confirmed (Figure 3 and Figure 4). The activation of PDH is achieved through PDH dephosphorylation catalyzed by PDH phosphatase (PDHP), which can be stimulated by calcium [17] (Figure 3A). Herein, we observed a decrease in the protein level of phosphorylated PDH (pPDH) and a decrease in pPDH fluorescence activity as a result of an increase in intracellular calcium levels (Figure 3B–D). The increased fluorescence activity of total PDH is shown in Figure S6. In hypoxic cancer cells, α-ketoglutarate (α-KG) can be converted to citrate in a reductive carboxylation reaction originated from the mitochondrial catabolism of glutamine. Herein, the restoration of the TCA cycle could alter the reductive carboxylation reaction even under hypoxia, starting with the activation of citrate synthase (Figure 4A). With or without glutamine, intracellular citrate level and isocitrate dehydrogenase activity were increased following activation of citrate synthase (Figure 4B,C, Figures S7 and S8). These results indicated that α-KG production was increased through the TCA cycle restoration rather than the reductive carboxylation reaction by glutamine under hypoxia (Figure 4D,E). Furthermore, following PDH knockdown (Figure 4F,G), the abundance of α-KG did not increase even after CaLac treatment, which confirmed the necessity for PDH activation in cancer cells for an increase of α-KG (Figure 4H).

### 3.3. Suppression of Hypoxia-Inducible Factor (HIF)-1α Transcriptional Activation by TCA Cycle Restoration

**Figure 5:**
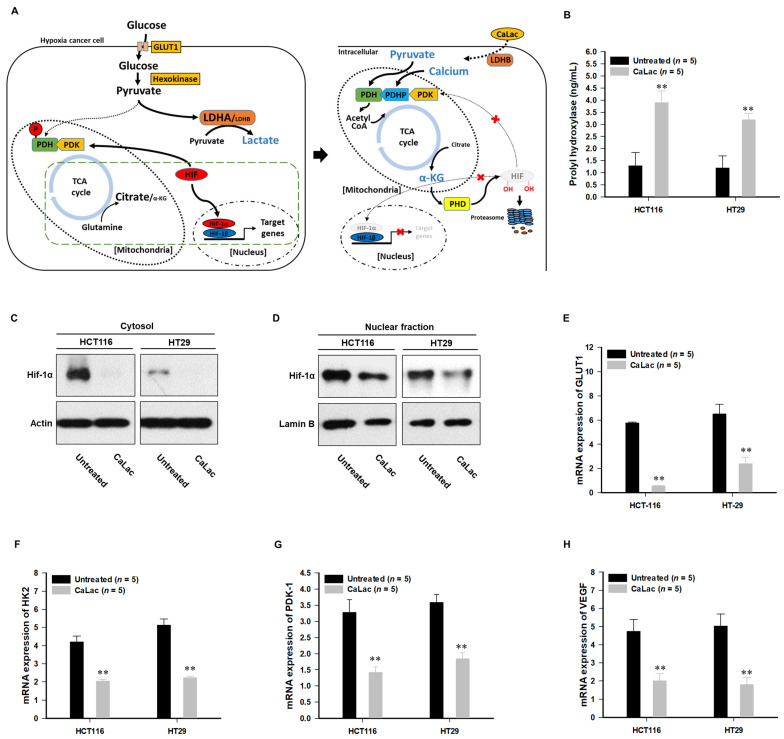
Inhibition of hypoxia-inducible factor (HIF)-1α transcriptional activity by an intermediate from the tricarboxylic acid (TCA) cycle. The enzymatic reaction of prolyl hydroxylase (PHD) was increased, leading to the proteasomal degradation of HIF-1α and decreased expression of oncogenes characteristic of HIF-1α-mediated transcriptional activation. (**A**) Schematic illustration of HIF-1α proteasomal degradation by alpha-ketoglutarate (α-KG), a TCA cycle intermediate. α-KG is produced as a metabolite, which acts as a substrate for PHD contributing to the proteasomal degradation of HIF-1a. (**B**) PHD enzymatic reaction following 2.5 mM CaLac treatment in CRC cells; 2 × 10^6^ cells were analyzed, respectively. (**C**,**D**) Proteasomal degradation of HIF-1α in the lysates of cytosol and nuclear fractions following 2.5 mM CaLac treatment in CRC cells. Uncropped blots were indicated in Figure S10. (**E**–**H**) Downregulation of glucose transporter (GLUT) 1, hexokinase (HK), pyruvate dehydrogenase kinase (PDK)-1, and vascular endothelial growth factor (VEGF) mRNA expression levels following 2.5 mM CaLac treatment in CRC cells. ** *p* < 0.001 vs. Untreated. Results are presented as mean ± standard deviation.

As PHD hydroxylates HIF-1α on two proline residues with co-factor α-KG and triggers proteasomal degradation [20], increased α-KG subsequent to TCA cycle restoration can be expected to inhibit HIF-1α-regulated oncogene expression (Figure 5A). Herein, we observed an upregulation of the PHD catalyzed enzymatic reaction subsequent to the increase in α-KG (Figure 5B), while HIF-1α was degraded at the protein level (Figure 5C,D). Decreased intracellular fluorescence activity of HIF-1α is shown in Figure S11. LDHB and PDH knockdown did not exhibit differences in HIF-1α expression, even after CaLac influx, demonstrating decreased HIF-1α expression following TCA cycle restoration (Figure S12). In response to HIF-1α suppression, we observed a decrease in the transcriptional activation of genes associated with the maintenance of anaerobic glycolysis. In addition to inhibiting the expression of GLUT1, the expression of hexokinase (HK) 2, the initial enzyme for catalyzing the phosphorylation of glucose, was also inhibited (Figure 5E,F). Furthermore, as the expression of PDK was remarkably reduced, an intracellular environment was established in which glycolysis and TCA cycle coupling were more activated as evidenced by decreased catalysis of PDH phosphorylation (Figure 5G). The expression of vascular endothelial growth factor (VEGF)-A was also significantly decreased following CaLac treatment (Figure 5H).

### 3.4. Anti-Cancer Effect on CRC and Suppressing Oncogene Expression Characterized by Hypoxia

**Figure 6:**
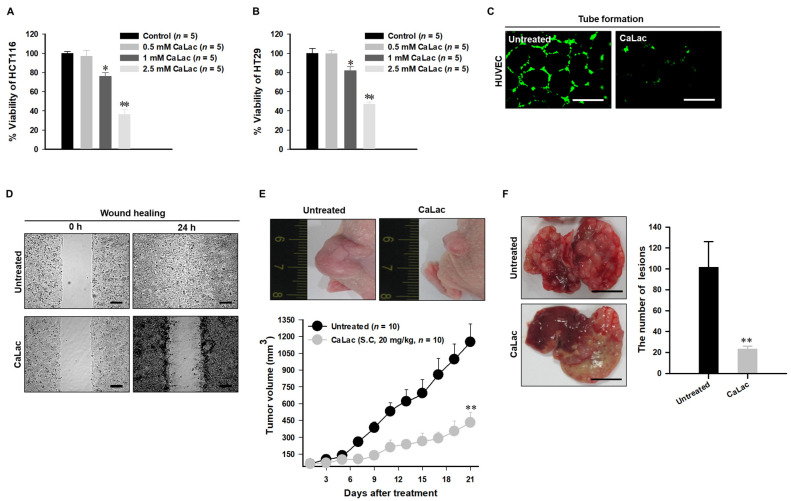
Confirmation of anti-cancer effect targeting human colorectal cancer (CRC). (**A**,**B**) The decrease in the CRC cell viability following dose-dependent CaLac treatment. (**C**) Inhibition of capillary-like tubular structures of human umbilical vein endothelial cells (HUVEC). Scale bars, 200 µm. (**D**) Inhibition of wound healing properties. ). Scale bars, 200 µm. (**E**) Anti-cancer effect on heterotopic xenograft model. (**F**) Anti-metastatic effect targeting the liver. Three individual samples were analyzed. Scale bars, 1 cm. ** *p* < 0.001 and * *p* < 0.05 vs. Untreated. Results are presented as mean ± standard deviation.

**Figure 7:**
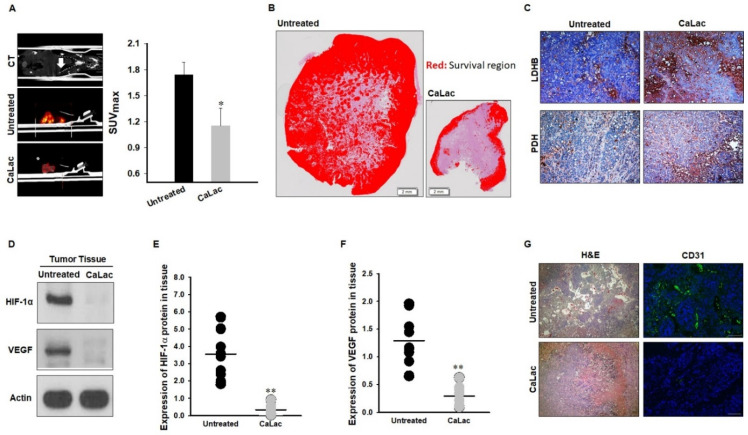
Confirmation of reduced malignancy following oncogene suppression. Proteasomal degradation of hypoxia-inducible factor (HIF)-1α inhibited the growth and migration of cancer cells through intensive changes in glycolytic metabolism in the hypoxic region. (**A**) Glucose uptake (standardized uptake value, SUVmax) in the fluorodeoxyglucose positron emission tomography/computed tomography (FDG-PET/CT). Quintuplicate analyses were performed. (**B**) Hematoxylin and eosin (H&E) staining-based identification of the survival region in tumor tissues. Scale bar, 2 mm. (**C**) Lactate dehydrogenase B (LDHB) and pyruvate dehydrogenase (PDH) expression in tumor tissues. Scale bar, 100 μm. (**D**–**F**) Protein expression levels of HIF-1α and vascular endothelial growth factor (VEGF)-A in tissue lysate. Ten individual samples were analyzed. Uncropped blots were indicated in Figure S15. (**G**) Comparison of vascularization (CD31) in tumor tissue between the untreated and CaLac-treated group. Scale bar, 100 μm. ** *p* < 0.001 and * *p* < 0.05 vs. Untreated. Results are presented as mean ± standard deviation.

The increased enzymatic activity of LDHB, restoration of the TCA cycle, and suppression of oncogenes via HIF-1α degradation contribute to the loss of malignancy in cancer cells while posing challenges for adaptation to hypoxia. As a result, the viability of CRC cells was significantly reduced by CaLac treatment (Figure 6A,B) while there was no toxicity on normal human colon fibroblast (Figure S13). Specifically, the suppression of VEGF-A (Figure 5H) directly affects the proliferation and migration properties of cancer cells. We confirmed that the angiogenic and invasiveness properties were decreased in the CaLac-treated CRC cells (Figure 6C,D). These characteristics are related to tumor progression, as tumor growth was reduced by approximately 80% in a heterotopic xenograft model with CRC cell transplantation in the left flank (Figure 6E). In the orthotopic model, polyp development was reduced by approximately 31% (Figure S14). Furthermore, the number of hepatic lesions caused by metastasis was also reduced by approximately about 80%, indicating the suppressed migratory capacity of CRC cells (Figure 6F). Additionally, the maximum standardized uptake value (SUVmax), as determined by FDG-PET/CT, was significantly decreased (Figure 7A). The survival region in the internal tumor was decreased, suggesting that the tumor failed to adapt to hypoxia (Figure 7B). Changes in the expression of LDHB and PDH were observed in tumor tissues as in CRC cells (Figure 7C). The protein expression levels of HIF-1α and VEGF-A were confirmed to be significantly downregulated in the tumor lysate (Figure 7D–F). The enzymatic activity of PHD was also increased in the tumor lysate, demonstrating an important role for PHD in the proteasomal degradation of HIF-1α (Figure S16). Moreover, the adequate formation of vasculature was not observed in tissues with decreased VEGF-A expression (Figure 7G).

## 4. Discussion

The introduction of CaLac into cancer cells is a deviation from conventional methods of inhibiting cancer progression that involve the suppression of metabolic enzymes in cancer cells [21]. This unique approach resulted in CaLac influx into CRC cells, activation of LDHB, and subsequent conversion of lactate into pyruvate (Figure 1, Figure 2, and Figure S3). These effects were primarily mediated by the lactate component of CaLac. Furthermore, calcium ions dissociated from CaLac played an essential role in the activation of PDH through inducing the dephosphorylation of PDH, which utilized pyruvate in the mitochondria (Figure 3 and Figure S6). These phenomena facilitated the restoration of the TCA cycle, which then inhibited the expression of hypoxia-associated genes, such as HIF-1α, and ultimately, abrogated cancer-specific metabolism under hypoxic conditions (Figure 4, Figure 5, and Figure S11) [22]. LDHB is an oxidoreductase that catalyzes the conversion of lactate into pyruvate, with the concomitant interconversion of NADH and NAD^+^ [13]. LDHB can be activated by lactate [23]; therefore, the influx of CaLac into human CRC cells could play a direct role in LDHB activation as a substrate (Figure 2). Following the knockdown of LDHB, pyruvate level did not change even following CaLac treatment, which strongly supported the notion that LDHB was activated following CaLac influx (Figure S4). However, in hypoxic cancer cells in which PDK is activated, PDH is inactivated in its phosphorylated form, thereby reducing pyruvate utilization in mitochondria [24]. In this condition, the role of calcium from CaLac is emphasized, as it has been well established that calcium is a strong activator of PDHP [25]. That is, CaLac contributes to the activation of PDH through the release of calcium in human CRC cells (Figure 3, Figure S6). α-KG is a key metabolic intermediate of the TCA cycle derived from isocitrate [26]. However, anaplerotic reactions can replenish components of the TCA cycle by synthesizing α-KG via reductive glutamine metabolism in cancer cells [27]. Glutamate oxaloacetate transaminase primarily converts glutamate into α-KG, thereby providing an additional energy source in cancer cells [27,28]. Therefore, it was necessary to determine whether the increased levels of α-KG were due to CaLac influx or the glutamine content of the medium. Results show that regardless of whether the medium contained glutamine or not, the concentration of α-KG was significantly increased by CaLac treatment (Figure 4). Moreover, the expression of citrate synthase and isocitrate dehydrogenase also increased, confirming that the increase in the levels of α-KG was due to the restoration of TCA cycle (Figure 4 and Figures S7 and S8). As the catalytic activity of PHD is suppressed under hypoxia, HIF-1α accumulates in the nucleus and dimerizes with HIF-1β to promote the transcription of hypoxia response genes [20]. However, HIF-1α stability depends on the TCA cycle intermediate, α-KG, which is a substrate for PHD [29]. Hence, an increase in the intracellular α-KG reverses HIF-1α stabilization through PHD hydroxylation [22]. HIF-1α is hydroxylated by PHD and then directed to the proteasome with the ubiquitin E3 ligase for degradation [30]. Thus, a decrease in HIF-1α expression was observed to downregulate the mRNA expression of hypoxia response genes in the current study, suggesting that cancer malignancy can be directly decreased (Figure 5 and Figure S11). We then tried to confirm whether CaLac influx could enhance the therapeutic potential in a xenograft mouse model established by human CRC cells (Figure 6 and Figure 7, and Figures S14 and S16). VEGF, one of the major target genes of HIF-1α, codes for a protein that stimulates tumor angiogenesis and neovascularization, which strongly correlates with cancer cell proliferation, migration, and metastasis [31,32]. In tumor-bearing mice administered CaLac by daily subcutaneous injection for 3 weeks, a decrease in tumor volume including the HIF1-α and VEGF deactivation, and suppression of liver metastasis were confirmed (Figure 6 and Figure 7). These in vivo antitumor effects were induced by specifically targeting the hypoxic region of the tumor. These results were supported by the observation of decreased glucose uptake and hypoxic cell death in the tumor.

## 5. Conclusions

Currently, targeting cancer-specific metabolism is a routine approach for inhibiting cancer progression, particularly via enzyme suppression [21]. However, the phenomenon of reversal of cancer-specific metabolism through LDHB activation is a unique method. Although our findings may be considered as a first step in impeding malignancy via remodeling cancer-specific anaerobic metabolism under hypoxia, they provided considerable insights into the supplementation of CaLac to patients with advanced CRC experiencing difficulties in treatment owing to the hypoxic tumor microenvironment.

## Figures and Tables

**Table 1:** Primer pairs were used for the quantitative real-time polymerase chain reaction (PCR).

Gene	Direction	Primer Sequences
*Actin*	Sense	5′-AACTGGAACGGTGAAGGT-3′
	Anti-sense	5′-CCTGTAACAACGCATCTCAT-3′
*VEGF*	Sense	5′-ACATCTTCCAGGAGTACCC-3′
	Anti-sense	5′-CTTGGTGAGGTTTGATCCG-3′
*PDK-1*	Sense	5′-TGTAGGTGGTATCATTCTCTTTC-3′
	Anti-sense	5′-GGATAACTAACAACACAGTCTCT-3′
*HK2*	Sense	5′-CAAAGTGACAGTGGGTGTGG-3′
	Anti-sense	5′-GCCAGGTCCTTCACTGTCTC-3′
*GLUT1*	Sense	5′-ACCACCTCACTCCTGTTA-3′
	Anti-sense	5′-CCACTTACTTCTGTCTCACTC-3′

## Data Availability

All collected data are included in this manuscript.

## Supplementary Materials

The following are available online at https://www.mdpi.com/2072-6694/13/7/1518/s1, Figure S1: In vivo study flow diagram. Figure S2: Ratio of lactate dehydrogenase (LDH) activity between A and B isoform. Figure S3: Separate confocal micrographs of lactate dehydrogenase (LDH) B expression for Figure 2B. Figure S4: Measuring pyruvate concentration following lactate dehydrogenase B (LDHB) knockdown. Figure S5: Uncropped blots and densitometry readings for Figure 3B. Figure S6: The increased fluorescence activity of total pyruvate dehydrogenase (PDH) by 2.5 mM lactate calcium salt (CaLac) treatment. Figure S7: Confirmation of intracellular citrate level following 2.5 mM lactate calcium salt (CaLac) treatment under normal (glutamine included) medium. Figure S8: Confirmation of intracellular isocitrate dehydrogenase activity following 2.5 mM lactate calcium salt (CaLac) treatment under normal (glutamine included) medium. Figure S9: Uncropped blots and densitometry readings for Figure 4G. Figure S10: Uncropped blots and densitometry readings for Figure 5C,D. Figure S11: The decreased fluorescence activity of hypoxia-inducible factor (HIF)-1α by 2.5 mM lactate calcium salt (CaLac) treatment. Figure S12: Confirmation of hypoxia-inducible factor (HIF)-1α expression in human colorectal cancer cells following lactate dehydrogenase (LDH) B or pyruvate dehydrogenase (PDH) knockdown. Figure S13: Confirmation of morphological change in human normal colon fibroblasts. Figure S14: Confirmation of the decreased in polyp development by lactate calcium salt (CaLac) administration in the orthotopic xenograft mouse model established by human colorectal cancer cells. Figure S15: Uncropped blots and densitometry readings for Figure 7D. Figure S16: Significantly increased expression of prolyl hydroxylase in tumor lysate following lactate calcium salt (CaLac) administration.
