# Supplementary material for: Remodeling of Cancer-Specific Metabolism under Hypoxia with Lactate Calcium Salt in Human Colorectal Cancer Cells
Source: Cancers (Basel). 2021 Mar 25;13(7):1518. doi: 10.3390/cancers13071518 (PMC8037473; doi:10.3390/cancers13071518)
Supplement: Supplementary file 1 [file cancers-13-01518-s001.pdf]

cancers

MDPI

Supplementary Materials: Remodeling of Cancer-Specific Metabolism under Hypoxia with Lactate Calcium Salt in Human Colorectal Cancer Cells

Keun-Yeong Jeong, Jae-Jun Sim, Min Hee Park, and Hwan Mook Kim

Supplementary Materials and Methods

1.1. Cell Lines and Culture Conditions

All cell lines were obtained from the American Type Culture Collection (ATCC). Human colon fibroblast cells (CCD-18Co) and colorectal cancer cells (HCT116 and HT29) were grown in RPMI1640 medium supplemented with 10% FBS and 1% penicillin/streptomycin. Human umbilical vein endothelial cells (HUVECs) were maintained using the EGM-2 bullet kit (Lonza, Basel, Switzerland). Cells were cultured under two conditions: a humidified atmosphere at 37 °C containing 5% CO_2, and a hypoxic culture was maintained at 1% oxygen, 5% CO_2 and 94% nitrogen for 24 h.

1.2. Reagents

Lactate calcium salt (CaLac) was purchased from Sigma (St. Louis, MO, USA). 2.5 mM lactate calcium salt was dissolved in distilled water. Aqueous lactate calcium salt was kept under 4 °C before treatment.

1.3. Quantification of Enzyme Levels

The cell culture medium was supplemented with CaLac, and cultures were incubated for 24 h; 2×10^6 cells were then sonicated. Lactate dehydrogenase A and B (LDHA and LDHB), isocitrate dehydrogenase, and prolyl hydroxylase activity were quantified using each assay kit (Biovision, Milpitas, CA, USA), according to the manufacturer's instructions following 2.5 mM CaLac treatment for 24 h under hypoxia.

1.4. Small Interfering RNA (siRNA) Transfection

Specific siRNAs were synthesized by Bioneer Corporation (Daejeon, Korea). The sequences of the primer pairs are as follows: siLDHB (sense: 5′-GUGAUUGGAAGUGGAUGUAdTdT-3′; anti-sense: 5′-UACAUCCACUUCCAAUCACdTdT-3′); siPDH (sense: 5′-CUGUACGCCGAAUGGAGUUdTdT-3′; anti-sense: 5′-AACUCCAUUCGGCGUACAGdTdT-3′). The siRNAs were transfected into CRC cells using RNAiMAX (Invitrogen, Carlsbad, CA, USA).

1.5. Western Blot Analysis

Protein lysates were prepared using a RIPA buffer protease inhibitor cocktail (Roche, Basel, Switzerland), and protein concentration were measured using the BCA assay kit (Thermo Scientific, Waltham, MA, USA). Proteins were separated on 6–12% sodium dodecyl sulfate polyacrylamide gel electrophoresis (SDS-PAGE) and transferred to polyvinylidene fluoride membranes. Specific primary antibodies included the following: GAPDH (1:10,000, Millipore, Burlington, MA, USA), α-tubulin (1:10,000, Millipore); Hif-1α (1:1000, BD Biosciences, San Jose, CA, USA); vascular endothelial growth factor (1:1000, BD Biosciences); Lamin B (1:1000, Santa Cruz Biotechnology, Dallas, TX, USA); LDHB (1:1000, Santa Cruz Biotechnology); PDH (1:1000, Cell Signaling, Danvers, MA, USA). Then, secondary antibodies were used for detecting specific proteins (1:10,000, Abclon, Seoul, Korea).

1.6. Cell Viability Assay

Colorectal cancer (CRC) cells were cultured in a 96-well plate (3 × 103 cells/well) for 24 h, and then the cells were treated with 2.5 mM CaLac for 24 h at 37 °C. Then, 10 μL of 3-(4,5-dimethylthiazol-2-yl)-2,5-diphenyltetrazolium bromide was added to each well, and the cells were incubated at 37 °C for 1 h in a humidified environment containing 5% CO_2. After the media was discarded, 200 μL of dimethyl sulfoxide (Cell Signaling Technology, Danvers, MA, USA) was added to each well. The absorbance was read at 570 nm using a microplate reader (iMark Microplate Absorbance Reader, Bio-Rad, CA, USA).

1.7. Immunocytochemistry

Colorectal cancer cells were fixed on the bio-coated coverslip (BD Bioscience, San Jose, CA, USA) using 4% paraformaldehyde. Cells fixed on coverslips were incubated for 15 h with the primary antibodies: lactate dehydrogenase B (LDHB, 1:200, Santa Cruz Biotechnology), pyruvate dehydrogenase (PDH, 1:200, Abcam, Cambridge, MA, USA), hypoxia-inducible factor-1α (Hif-1α, 1:200, Novus Biologicals, Centennial, CO, USA). Then, coverslips were incubated with anti-rabbit secondary biotinylated antibody and visualized with streptavidin conjugated to Fluorescein (Vector). Quantitative signal intensity was analyzed using Xenogen In Vivo Imaging System (IVIS® 100 series, Caliper Life Science, Waltham, MA, USA).

1.8. Metabolite Assay

The cell culture medium was supplemented with CaLac, and cultures were incubated for 24 h; 2 × 106 cells were then sonicated and passed through a 10 kDa centrifuge filter (Biovision, Milpitas, CA, USA) at 12,000× *g* for 10 min. Citrate was quantified using citrate assay kit (Biovision, Milpitas, CA, USA), following the manufacturer's protocol.

1.9. Orthotopic Xenograft Model

The Association of Assessment and Accreditation of Laboratory Animal Care approved the animal facilities, and all experiments were performed by conforming with the guidelines established by the Institutional Animal Care and Use Committee at Gachon University (IACUC-LCDI-2019-0102). Ten balb/c nude mice (5 weeks of age) were purchased from Charles River Breeding Laboratories (Wilmington, MA, USA). All animals were maintained in a 12 h light/dark cycle (light on, 08:00) at 22–25 °C with free access to food and water. To establish the orthotopic xenograft model, HCT-116 cells (1×10^4) were suspended in 10 μL of phosphate-buffered saline and injected in the cecal region of the mice. After health restoration, mice were subcutaneously administered with 20 mg/kg CaLa daily for 21 days. On the last day of the experiment, all mice were euthanized with carbon dioxide inhalation, and macroscopic analyses were performed.

1.10. Statistical Analysis

All data are presented as mean ± standard deviation. Statistical significance was analyzed using the Student's *t*-test or the one-way analysis of variance (ANOVA), depending on the normality of the data. A difference of $p < 0.05$ was considered to be statistically significant. All statistical analyses were carried out using Sigma Stat (ver. 3.5, Systat Software Inc., San Jose, CA, USA).

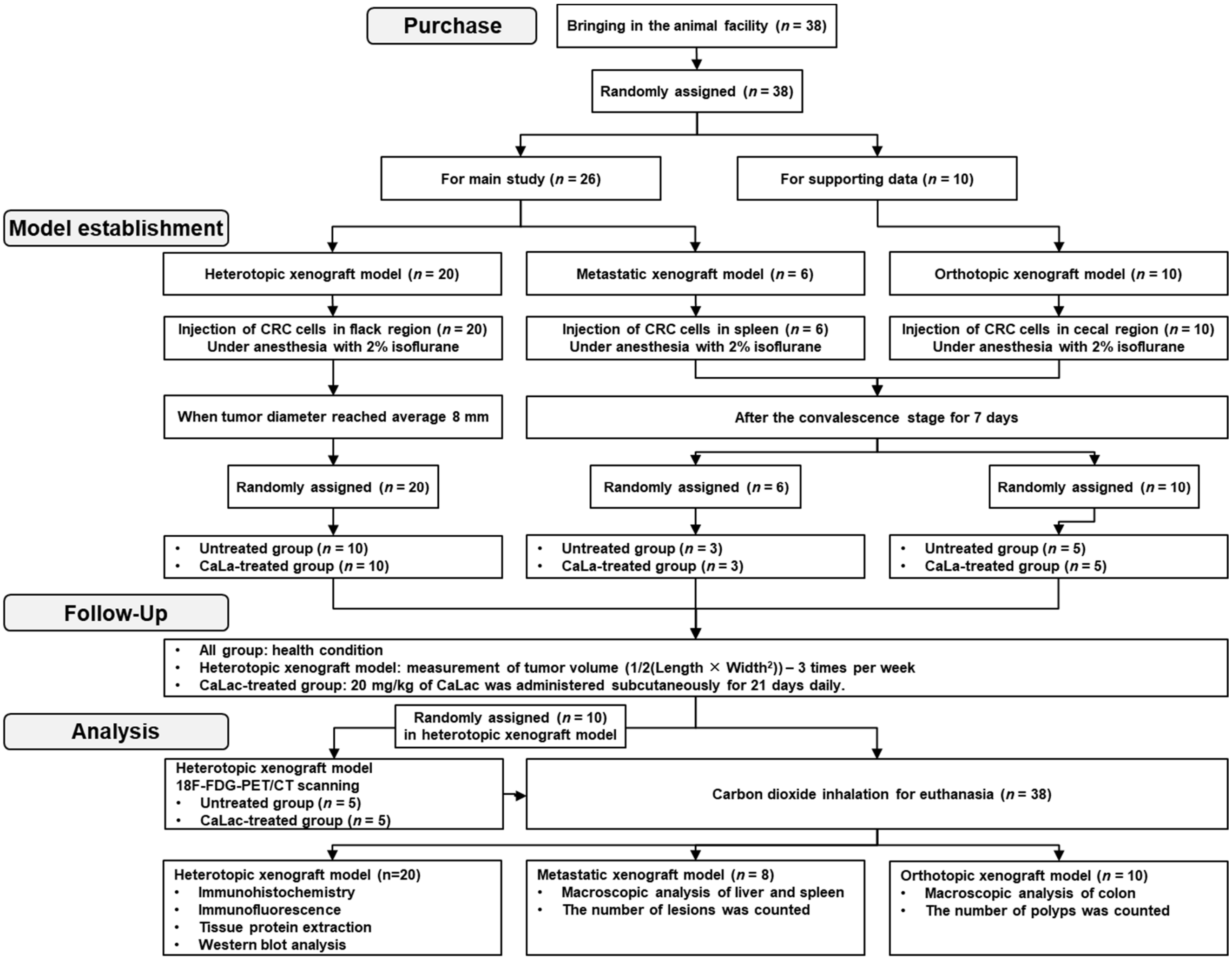

Figure S1. In vivo study flow diagram. Thirty eight heads of balb/c nude mice were purchased and assigned to heterotopic xenograft model (n = 20), metastatic xenograft model (n = 8), and orthotopic xenograft model (n = 10). No object was excluded during the establishment of each model. Following the establishment of each xenograft model by human colorectal cancer (CRC) cells, the total mice per each model were assigned equally to the untreated group and the lactate calcium salt (CaLac)-treated group. In the heterotopic xenograft model, 10 mice were randomly assigned for fluorine-18-fluorodeoxyglucose positron emission tomography/computed tomography (18F-FDG-PET/CT) scanning (untreated group, n = 5; CaLac-treated group, n = 5). The mice that completed 18F-FDG-PET/CT scanning returned to their cages. Finally, all mice were euthanized with carbon dioxide inhalation for analysis. Histological and molecular biological analyses were performed on a heterotopic xenograft model (n = 20). Macroscopic analyses were performed on the metastatic (n = 8) and orthotopic xenograft models (n = 10).

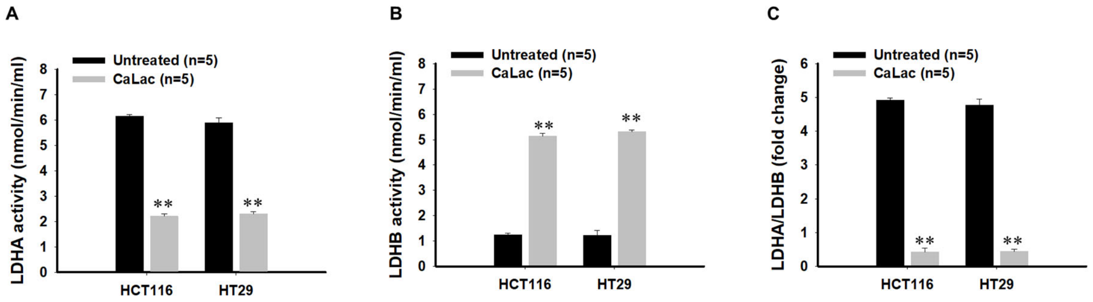

Figure S2. Ratio of lactate dehydrogenase (LDH) activity between A and B isoform. (**A**) Decrease in the LDHA activity following 2.5 mM lactate calcium salt (CaLac) treatment in colorectal cancer (CRC) cells under hypoxia. (**B**) Increase in the LDHA activity following 2.5 mM CaLac treatment in CRC cells under hypoxia. (**C**) Decrease in the LDHA/LDHB ratio. ***p* < 0.001 vs. Untreated. Results are represented as mean ± standard deviation.

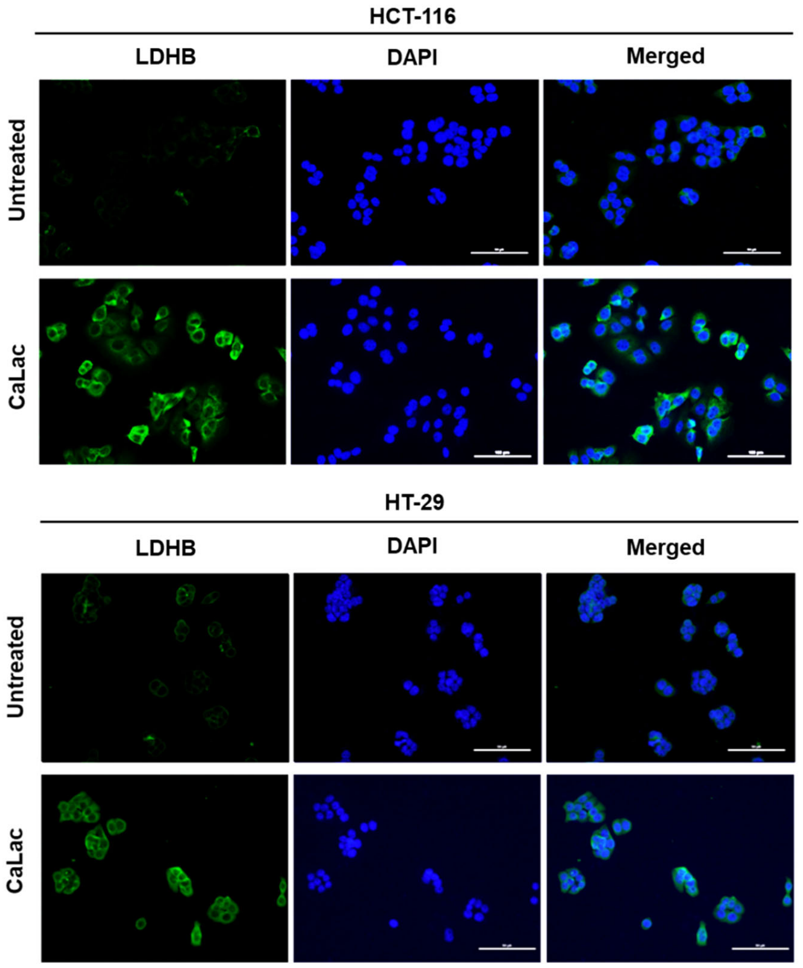

Figure S3. Separate confocal micrographs of lactate dehydrogenase (LDH) B expression for Figure 2B. 2.5 mM lactate calcium salt (CaLac) was treated in colorectal cancer cells for 24 h. Scale bars, 100 μm.

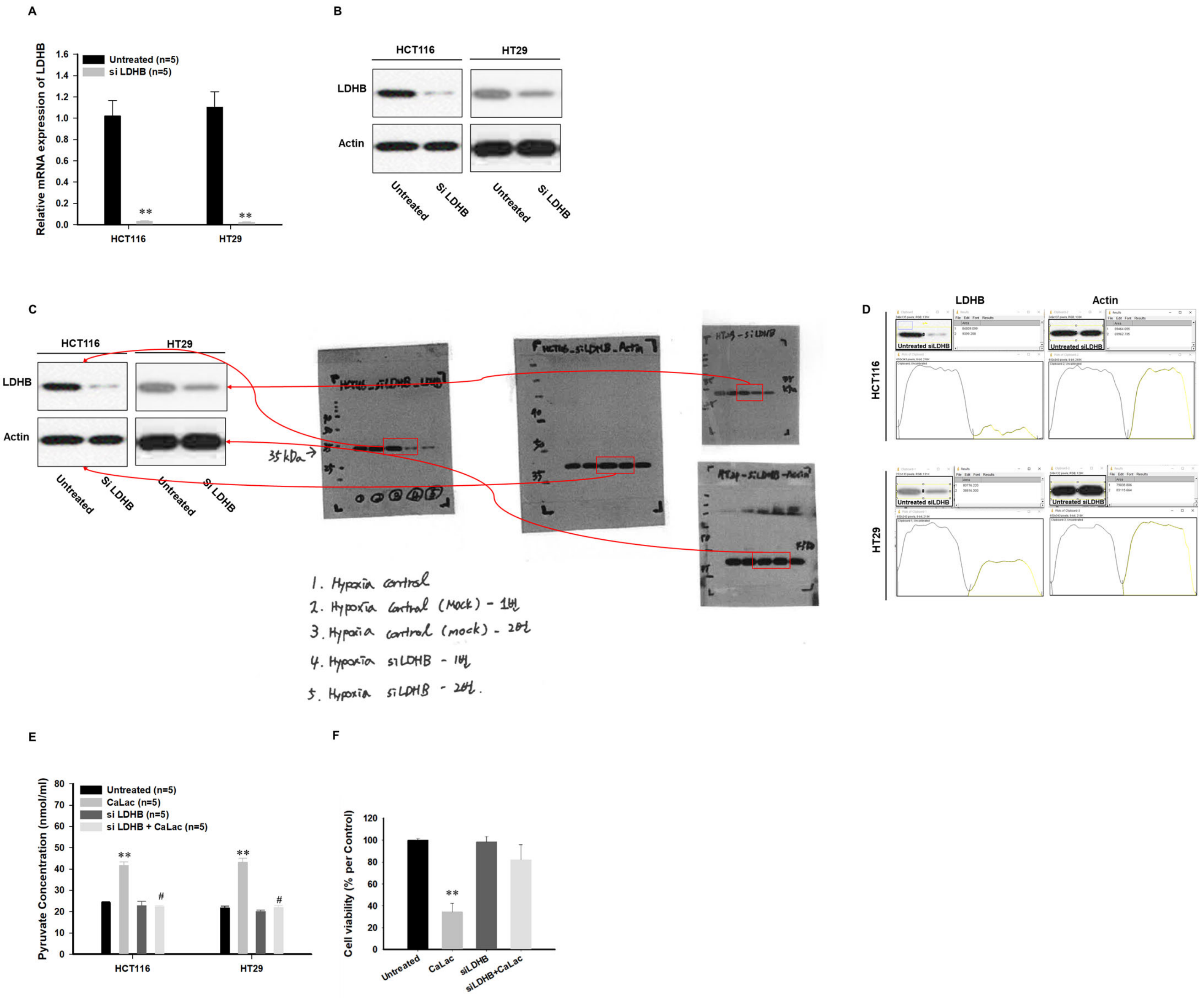

Figure S4. Measuring pyruvate concentration following lactate dehydrogenase B (LDHB) knockdown. (**A** and **B**) Confirmation of LDHB knockdown condition by mRNA and protein expression in colorectal cancer (CRC) cells following siLDHB transfection. (**C**) Uncropped blots of Figure S4B. (**D**) Densitometry readings for blots in Figure S4B. (**E**) Comparison of pyruvate production in LDHB knockdown CRC cells following 2.5 mM CaLac treatment. (**F**) Comparison of CRC cell viability following LDHB knockdown. **$p < 0.001$ vs. Untreated. #$p < 0.001$ vs CaLac. Results are represented as mean ± standard deviation.

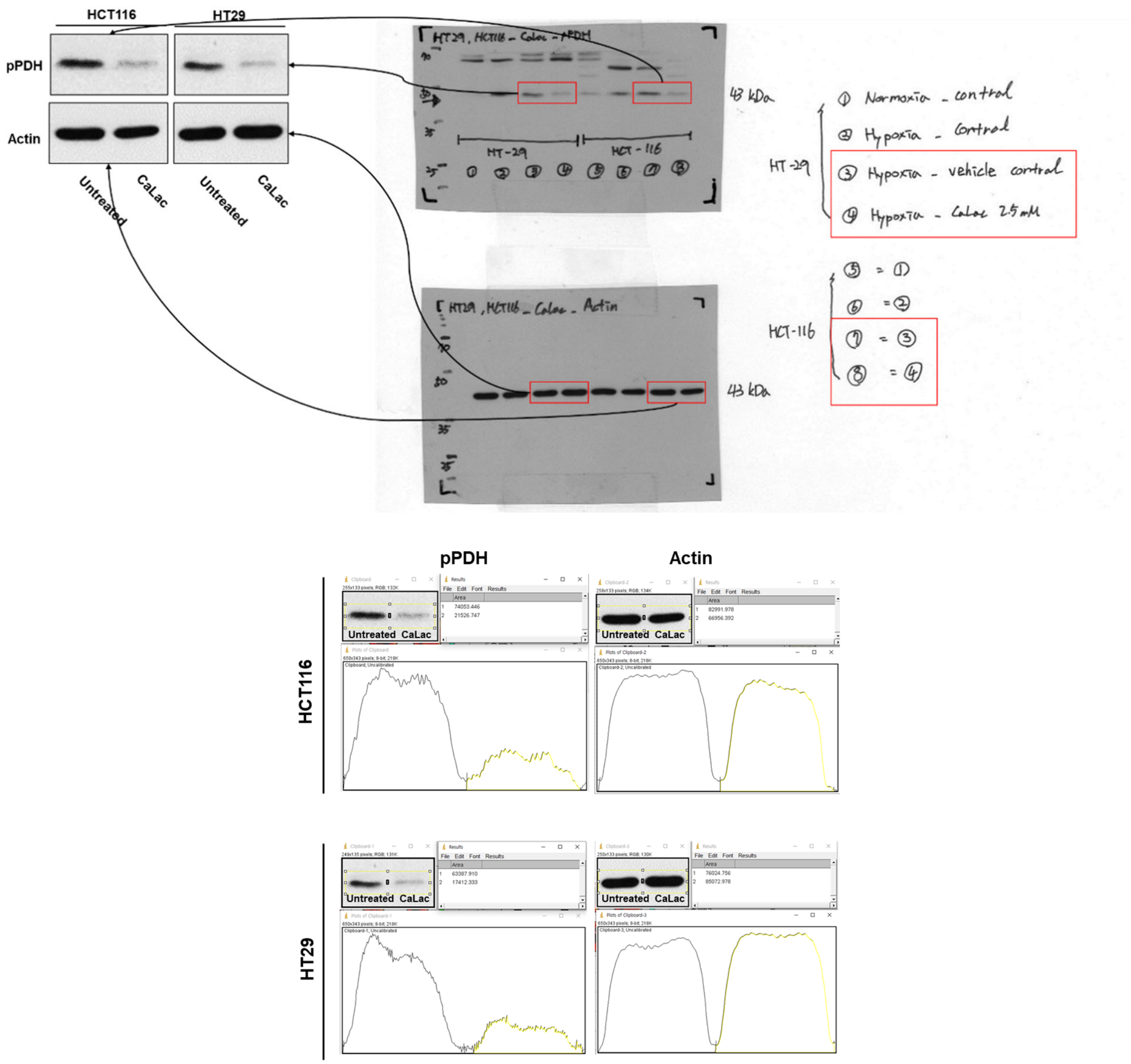

Figure S5. Uncropped blots and densitometry readings for Figure 3B.

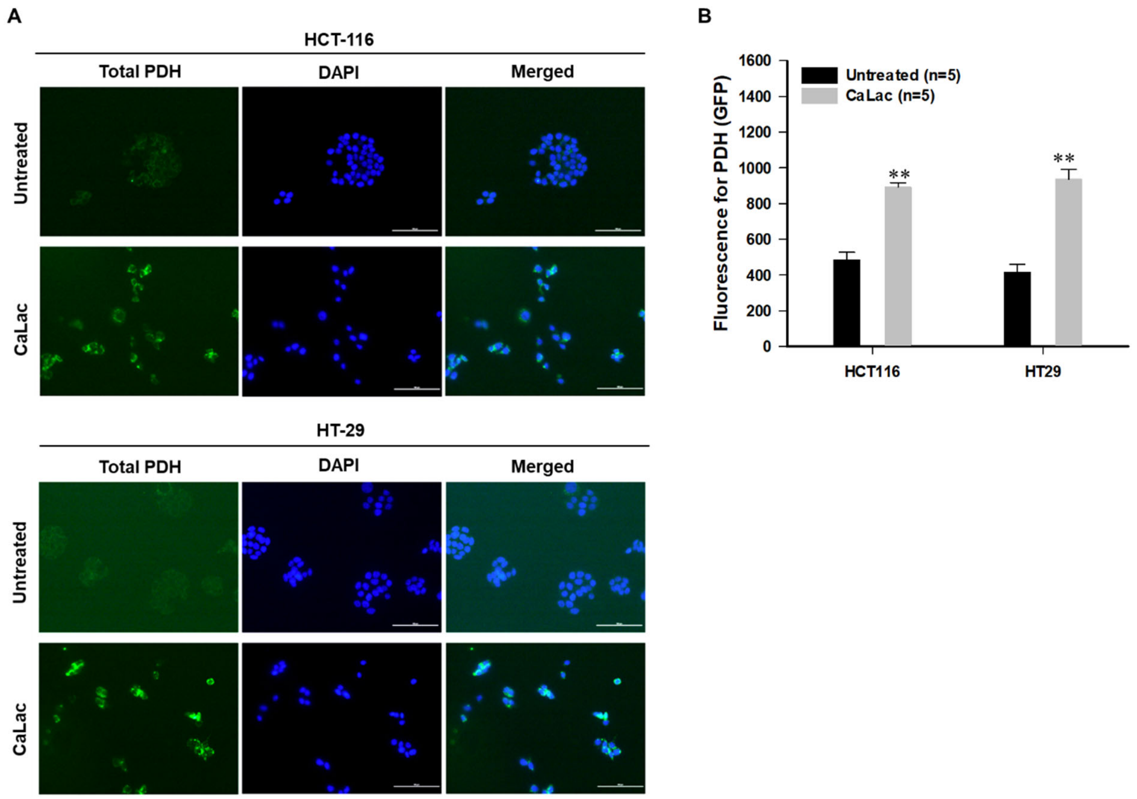

Figure S6. The increased fluorescence activity of total pyruvate dehydrogenase (PDH) by 2.5 mM lactate calcium salt (CaLac) treatment. (**A**) Confocal micrographs of total PDH activation in colorectal cancer cells. Scale bars, 100 µm. (**B**) Quantitative analysis for the PDH fluorescence (GFP) intensity. ** $p < 0.001$ vs. Untreated. Results are represented as mean ± standard deviation.

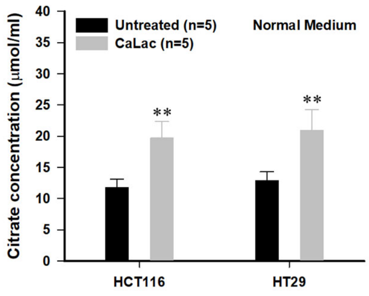

Figure S7. Confirmation of intracellular citrate level following 2.5 mM lactate calcium salt (CaLac) treatment under normal (glutamine included) medium. ** $p < 0.001$ vs. Untreated. Results are represented as mean ± standard deviation.

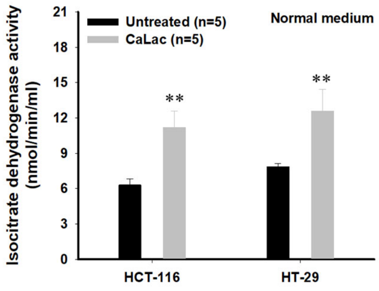

Figure S8. Confirmation of intracellular isocitrate dehydrogenase activity following 2.5 mM lactate calcium salt (CaLac) treatment under normal (glutamine included) medium. $^{**}p < 0.001$ vs. Untreated. Results are represented as mean ± standard deviation.

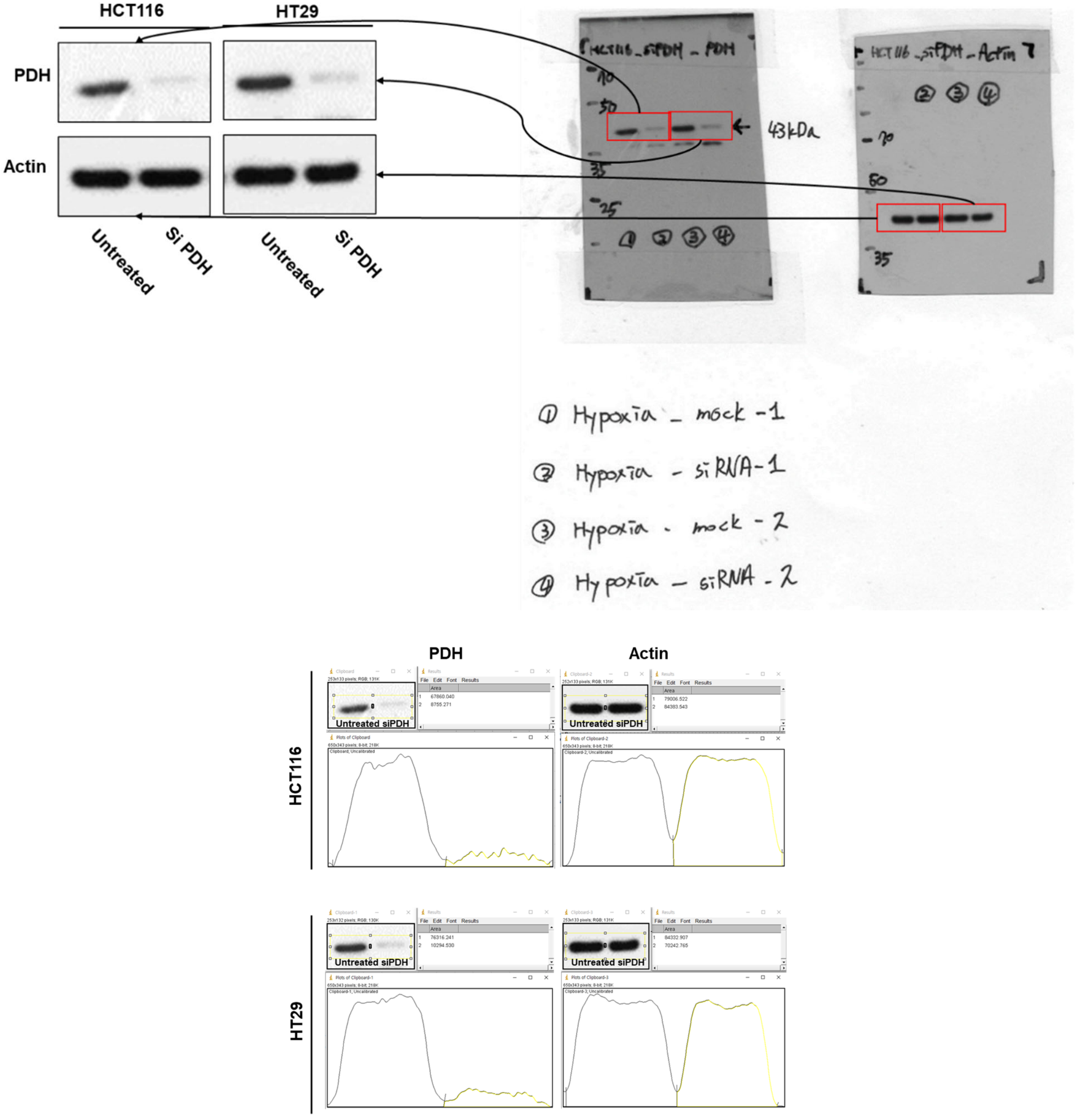

Figure S9. Uncropped blots and densitometry readings for Figure 4G.

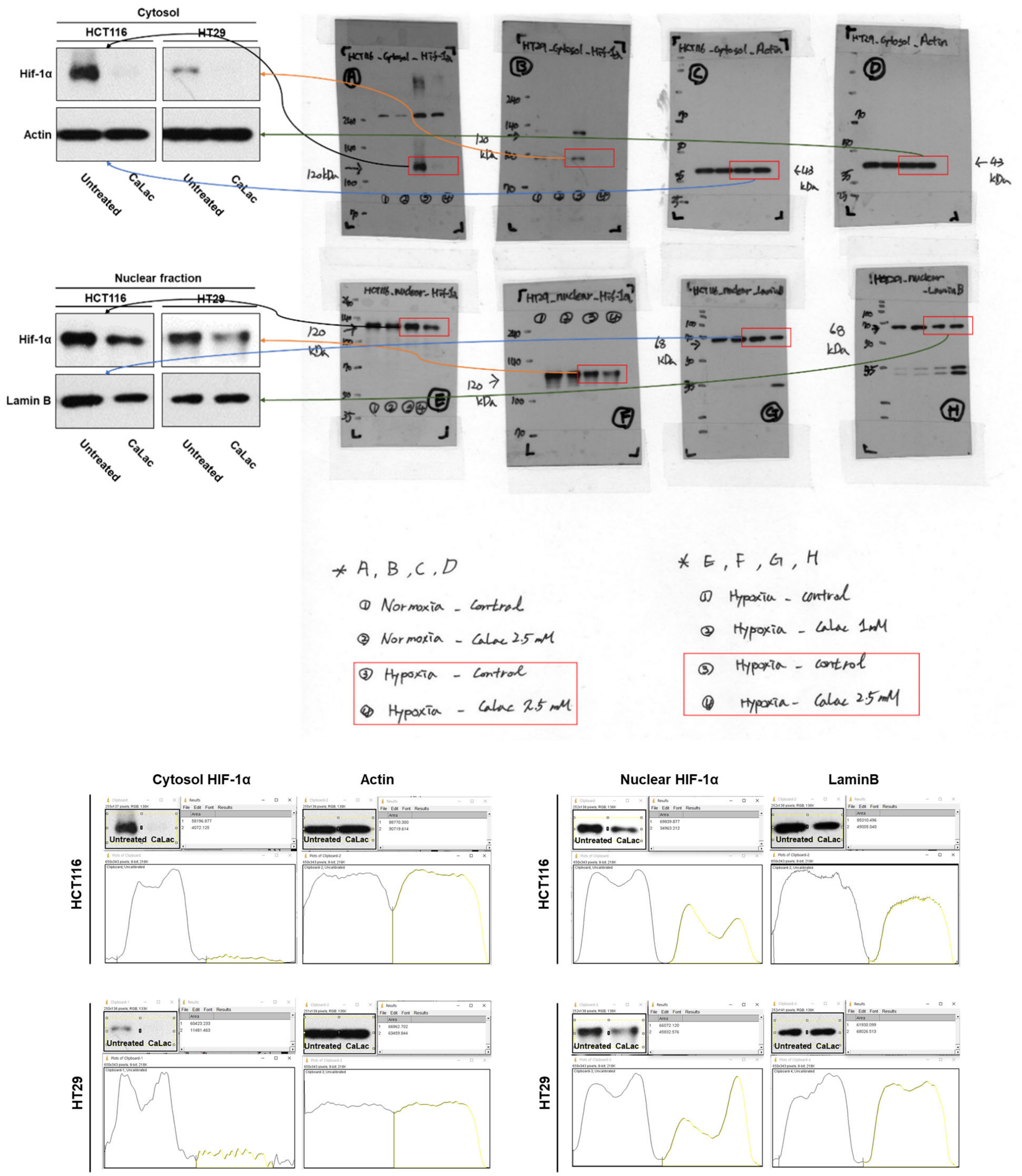

Figure S10. Uncropped blots and densitometry readings for Figure 5C and D.

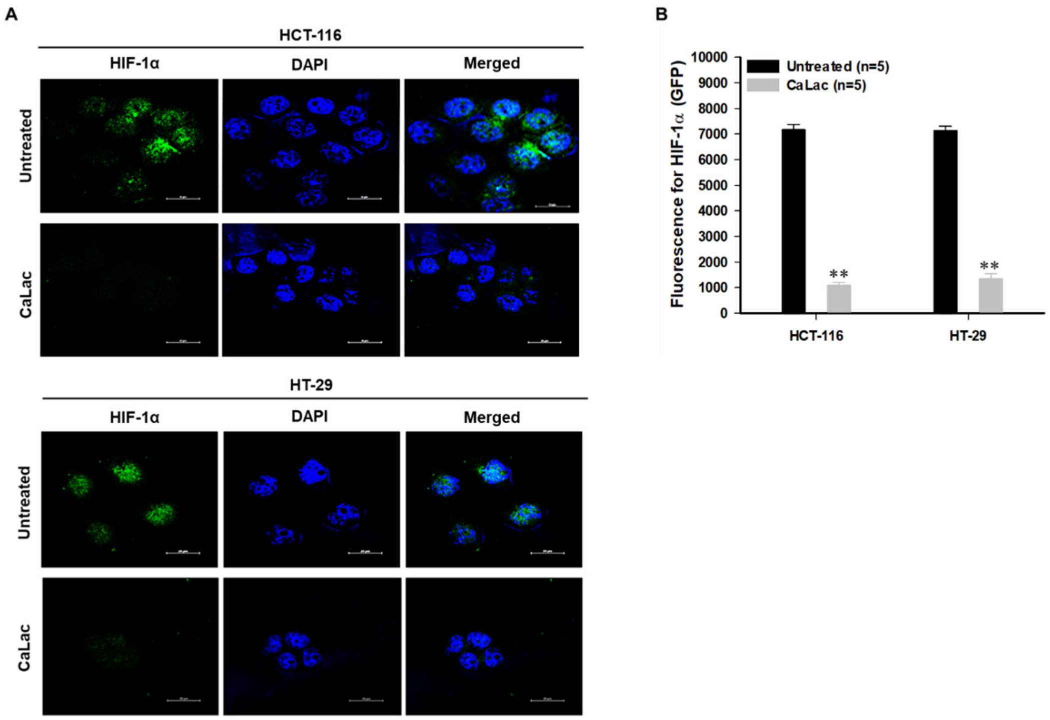

Figure S11. The decreased fluorescence activity of hypoxia-inducible factor (HIF)-1*α* by 2.5 mM lactate calcium salt (CaLac) treatment. (**A**) Confocal micrographs of HIF-1*α* suppression in colorectal cancer cells. Scale bars, 100 μm. (**B**) Quantitative analysis for the HIF-1*α* fluorescence (GFP) intensity. $^{**}p < 0.001$ vs. Untreated. Results are represented as mean ± standard deviation.

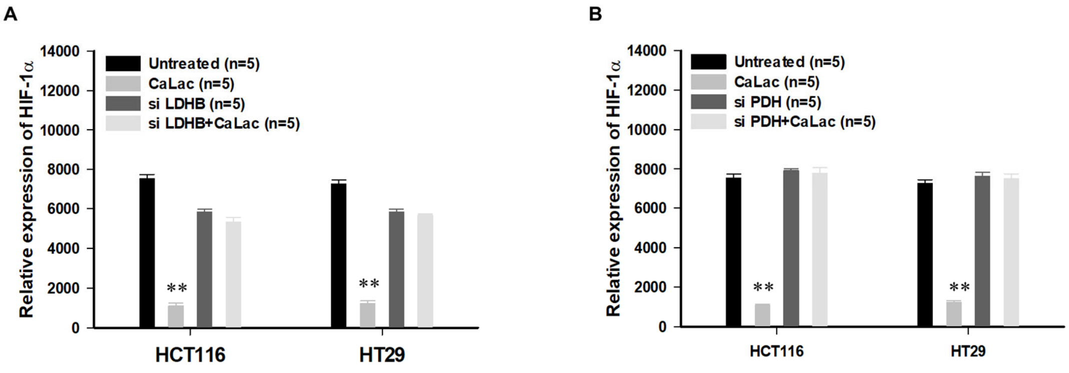

Figure S12. Confirmation of hypoxia-inducible factor (HIF)-1*α* expression in human colorectal cancer cells following lactate dehydrogenase (LDH) B or pyruvate dehydrogenase (PDH) knockdown. (**A**) Comparison of HIF-*α* expression among the groups following LDHB knockdown. (**B**) Comparison of HIF-*α* expression among the groups following PDH knockdown. 2.5 mM of CaLac was treated in the CaLac and si PDH+CaLac groups. $^{**}p < 0.001$ vs. Untreated. Results are represented as mean ± standard deviation.

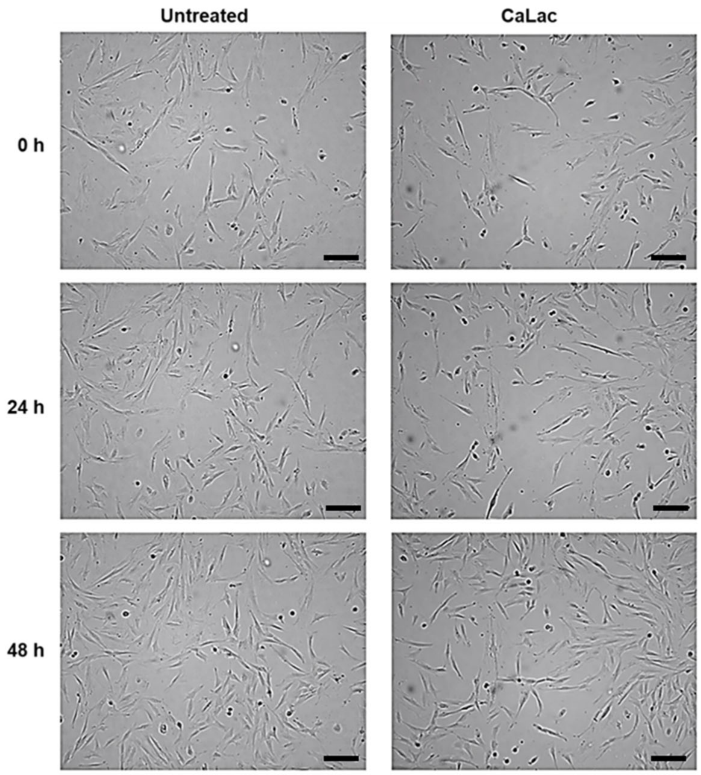

Figure S13. Morphological proof of the lacking the effect on the human normal colon fibroblasts following 2.5 mM lactate calcium salt (CaLac) treatment. The results show normal proliferation of the colon fibroblast even after 2.5 mM CaLac treatment. Scale bars, 250 μm.

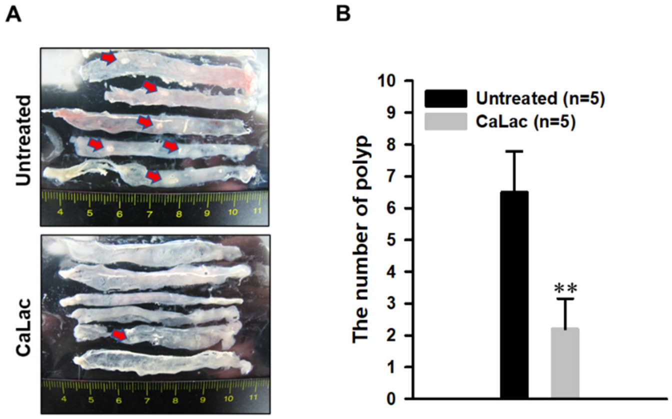

Figure S14. Confirmation of the decreased in polyp development by lactate calcium salt (CaLac) administration in the orthotopic xenograft mouse model established by human colorectal cancer cells. (**A**) Representative pictures for intestinal polyp development. (**B**) Quantitative analysis for polyp development between the untreated and CaLac-treated group. ** $p < 0.001$ vs. Untreated. Results are represented as mean ± standard deviation (SD).

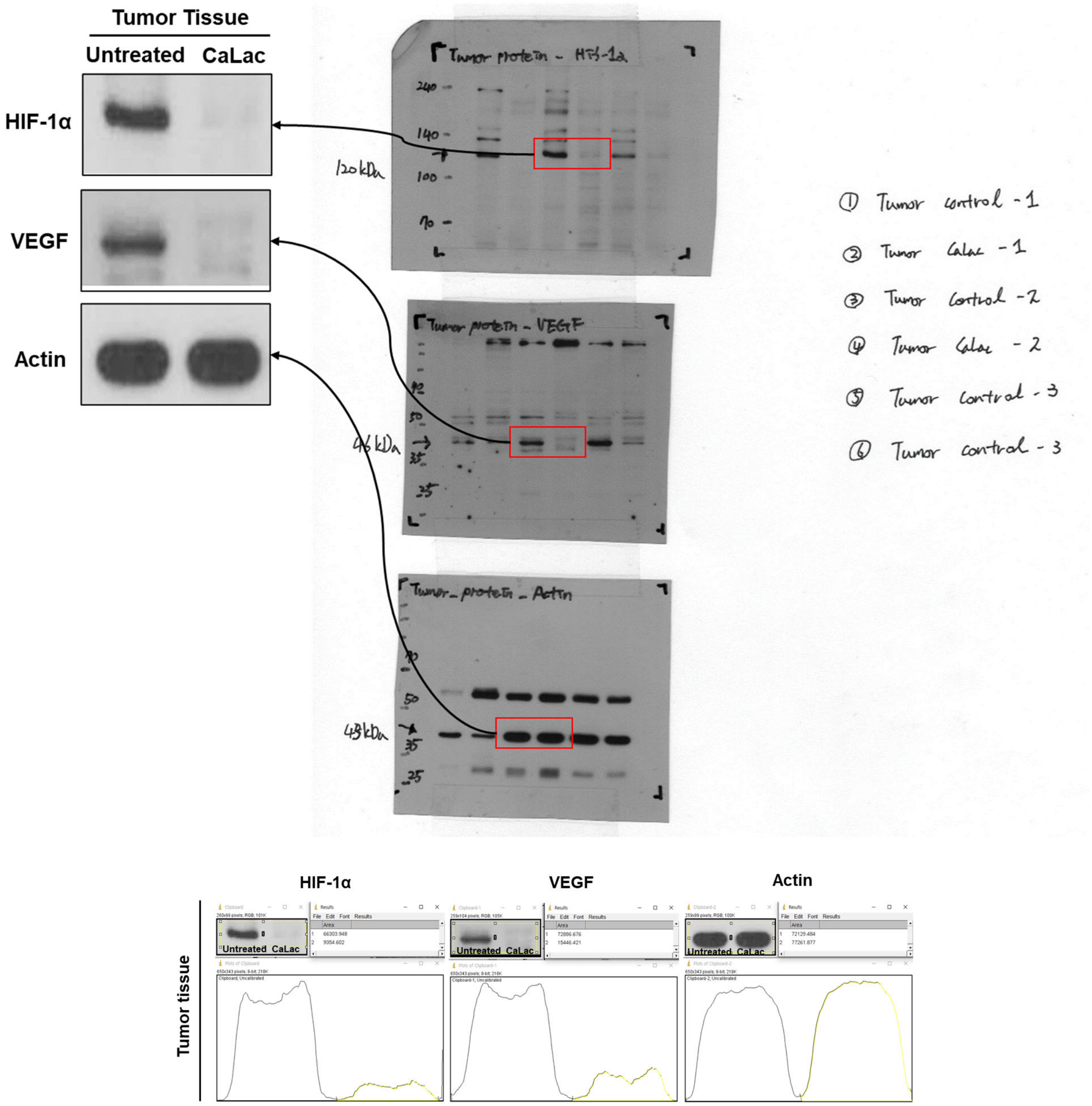

Figure S15. Uncropped blots and densitometry readings for Figure 7D.

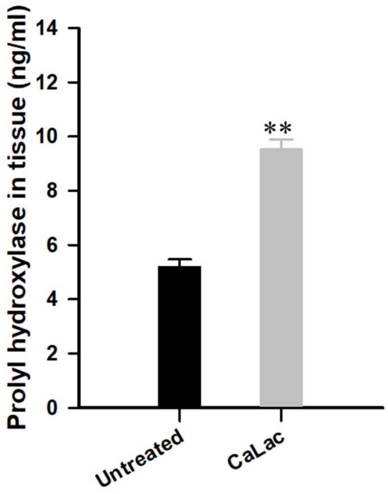

Figure S16. Significantly increased expression of prolyl hydroxylase in tumor lysate following lactate calcium salt (CaLac) administration. Quintuplicate analyses were performed. ***p* < 0.001 vs. Untreated. Results are represented as mean ± standard deviation.
